# Livestock exposure to future cumulated climate-related stressors in West Africa

**DOI:** 10.1038/s41598-022-22544-y

**Published:** 2023-02-15

**Authors:** Audrey Brouillet, Benjamin Sultan

**Affiliations:** ESPACE-DEV, Univ Montpellier, IRD, Univ Guyane, Univ Reunion, Univ Antilles, Univ Avignon, Maison de la Télédétection, 500 rue Jean-François Breton, F-34093 Montpellier, Cedex, France

**Keywords:** Climate-change impacts, Projection and prediction

## Abstract

A large range of climate change impacts is expected during the twenty-first century in vulnerable regions such as West Africa, where local populations largely rely on livestock systems as their main food production and income source. As climate change threatens livestock systems in various ways, here we assess how regional livestock could be exposed to cumulated climate-related stressors in the future. Using the world’s largest multi-model climate impacts simulations database ISIMIP, we find that a large part of West Africa will experience at least 5–6 cumulated multiple climate stressors before the 2030s, including amplified severe heat stress conditions and flood risks. Consequently, about 30% of the current total regional livestock could be exposed to these cumulated stressors, with sheep and goat as the most affected species. This paper brings new quantifications that may help policy makers to prioritize decisions that could prepare local populations to face multiple climate-related impacts.

## Introduction

Mean global temperatures are projected to continue rising according to Earth System Models, as an unequivocal consequence of human-induced increase of greenhouse gases concentration. Previous papers have robustly exhibited a large range of multiple resulting impacts on specific global and regional attributes, both at historical and future time scales^1–4^. In the future, these impacts will emerge and intensify heterogeneously across the world, and affect multiple sectors including water ressources, agriculture, health, and climate extreme events^5–7^.

In such key sectors as food sustainability, future projections of combined global impact models (GIMs) and Earth System Models (ESMs) show that climate change will increase the number of people at risk of hunger by 8 to 80 million people by 2050 at global scale, with 80% expected in Asia and Africa^8–10^. Accordingly, livestock systems and food production sources will be negatively affected due to rising temperatures^10–12^, soil degradation and soil organic matter loss^13,14^. Increasing temperatures will also increase animal water need that might further result in a reduced access to sources of drinking water for animal husbandry^15^. Models emphasize that the livestock ability to support both livelihood and the increasing demand for productivity could be threatened with various intensities depending on the region and the warming level^16^. Nevertheless, only few studies have investigated the livestock exposure to multiple climate-related and cross-sectoral risks or stressors resulting from global warming^17,18^, and particularly how they might cumulate in space and co-occur in time within vulnerable regions. Yet, multiple risks relying on future climate evolution may threaten regional nutrition and livelihoods of millions of people, especially in low-income countries where people directly rely on livestock productivity for food security^10^.

This paper characterizes how major livestock species could be exposed to cumulated climate-related stressors during the 21st century in West Africa. In this study, a stressor is defined as an adverse evolution of a climate-related indicator that may unfavorably affect animals. We investigate how future multiple climate stressors will whether cumulate and worsen regional livestock exposure to severe risks under a continuous global warming, or if projected changes of such stressors could mute themselves depending on the location. A cumulative approach based on the significance of a given projected change is used, and we analyse a set of eight various indicators that could affect livestock in the future. These indicators include hydrological extremes, heat induced stress on livestock and available vegetal cover for grazing. In order to provide the largest range of combined climate and impact responses for each indicator and account for associated uncertainties, the highest number of corresponding GIMs $\times$ ESMs simulations is analysed under the highest warming scenario (RCP8.5^19^) from the Inter Sectoral Impact Model Intercomparison Project phase 2b (ISIMIP2b)^20^.

## Results

### Future projections of multiple climate-related indicators

**Figure 1:**
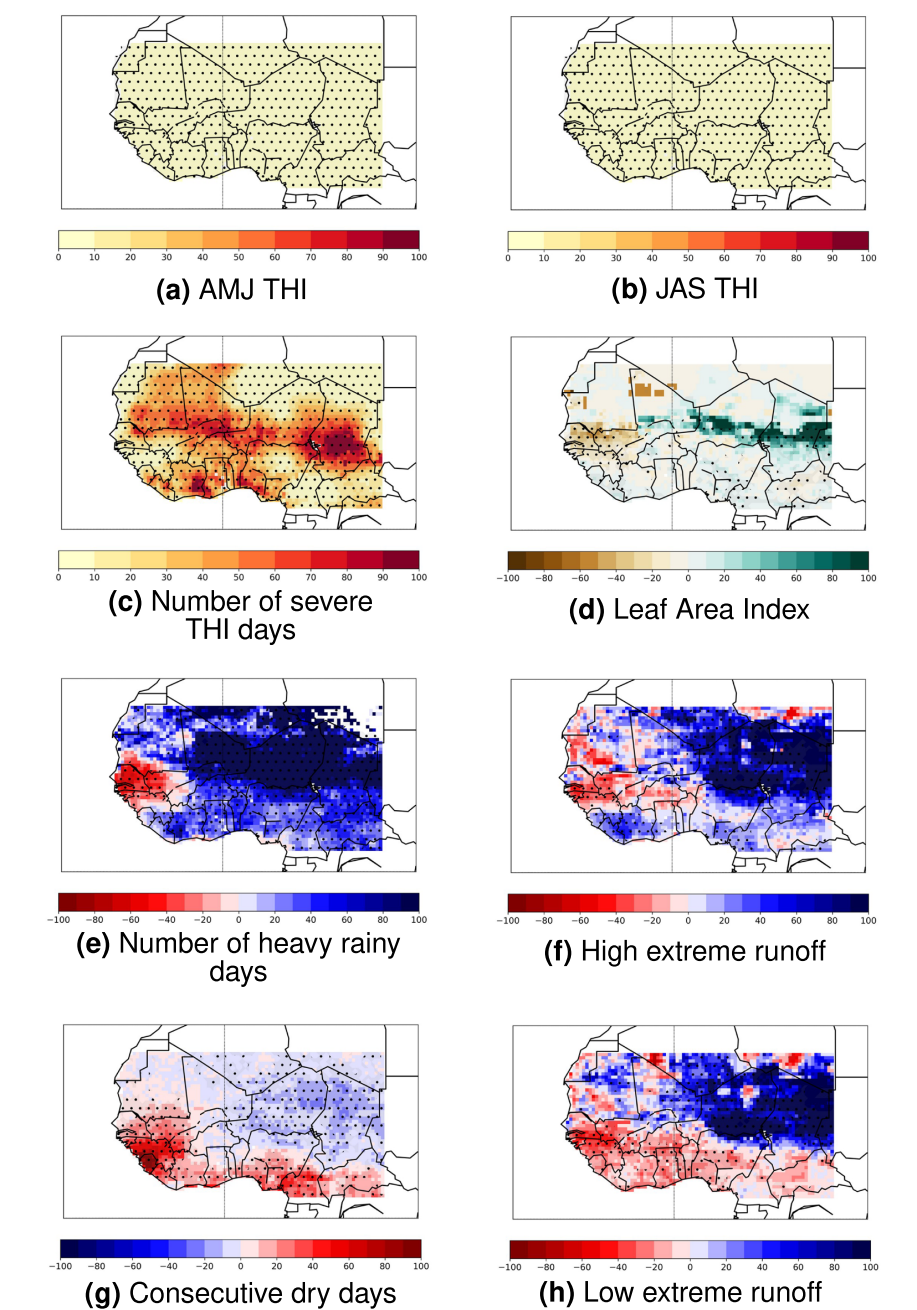
Spatial distribution of multi-model mean projected changes between 1979–2005 and 2074–2100 under the RCP8.5 scenario for the eight selected climate stressor indicators over Western Africa in $\%$ of historical values (see Figure S1). **(a)** April-to-June mean THI (no unit). **(b)** July-to-September mean THI (no unit). **(c)** Number of days per year with *severe* daily heat stress (THI $\ge$ 89). **(d)** Annual mean Leaf Area Index (no unit). **(e)** Number of days per year with daily precipitation larger than 20mm. **(f)** Annual 98th percentile of surface + subsurface runoff (i.e. high flow index, in mm/day). **(g)** Number of days per year with daily precipitations smaller than 3mm. **(h)** Annual 2nd percentile of surface + subsurface runoff (i.e. low flow index, in mm/day). Dots show grid points where at least 50$\%$ of the models agree on the sign of the change. Details about total number of simulations used per impact indicator is displayed in Table S1.

Under the RCP8.5, patterns of changes of April-to-June and July-to-September mean heat stress (illustrated by the Temperature-Humidity Index, THI^21,22^) show seasonal intensifications of +4–6 units between 1979–2005 and 2074–2100 over West Africa (Fig. 1a,b). These increasing patterns are quite spatially homogeneous, except over the west coast for both seasons and over the north-east for AMJ. These patterns are consistent with previous studies about the heat stress over this region^22,23^. A small increase in *severe* heat stress days (i.e. days per year with THI $\ge$ 89^22^) is shown over the west coast, north-east and south-east of West Africa, whereas locations along 10–12$^{\circ }$N, Ivory Coast and southern Nigeria are characterized by a large future intensification of at least 3 months more per year of severe heat stress conditions (Fig. 1c). These spatial discrepancies can be explained by the spatial heterogeneity of the future changes in relative humidity (RH) over West Africa, as models project that RH will whether dampen or emphasize heat stress increases depending on the local RH change sign^23,24^. Severe heat stress spatial differences may also result from the threshold effect of this metric, since severe heat stress days are detected above a given THI value that might be not enough or too much captured by models. Annual mean Leaf Area Index (LAI) is here analysed as a vegetal cover proxy, and shows a dipole of future decrease in the western part and an increase in the eastern part of the region, with some local decrease/increase patterns (Fig. 1d). Figure 1e–h all display a significant dipole of future wettening of the eastern of West Africa and a drying in the western part, consistently with previous studies^25–27^. This hydrological dipole (wettening vs drying) coincides with future projected LAI changes (increase vs decrease), particularly the high surface runoff metric (Fig. 1d,f). Since GIMs assessed for the LAI differ from GIMs analysed for surface runoff metrics, but the four ESMs used as climate input for the two impact metrics are the same (Table S1), our results suggest that input precipitations for impact modelling are the the main driver of LAI and runoff output simulations (Figure S3). It also shows a good mean representation between the 10 selected CMIP5 ESMs (Fig. 1e,g) and the four ESMs used in ISIMIP2b (Figure S3). These patterns emphasize that GIMs $\times$ ESMs simulations exhibit a good robustness per indicator in multi-model mean, except for specific area/indicator combinations, e.g. the projected drying over the western part of the region (Fig. 1d–h).

Projected decrease and increase both depict an adverse or a positive future scenario depending on the climate-related indicator. For example, a decrease in annual mean LAI illustrates less food available for livestock in the future, whereas a LAI increase depicts more vegetal cover and graze availability^8,28,29^. In the following cumulated stressors analysis, the *adverse scenario* is investigated, that is the scenario in which future changes in climate-related indicators can be considered as *stressors* due to worsened evolution. This adverse scenario can be illustrated by: a future increase in the three heat stress indicators, an annual mean LAI decrease, a consecutive dry days increase, a low runoff decrease, and heavy rainy days and high runoff increases. Same analysis are conducted for the corresponding *positive scenario* and displayed in Supplementary Information.

### Cumulated climate stressors and livestock exposure

**Figure 2:**
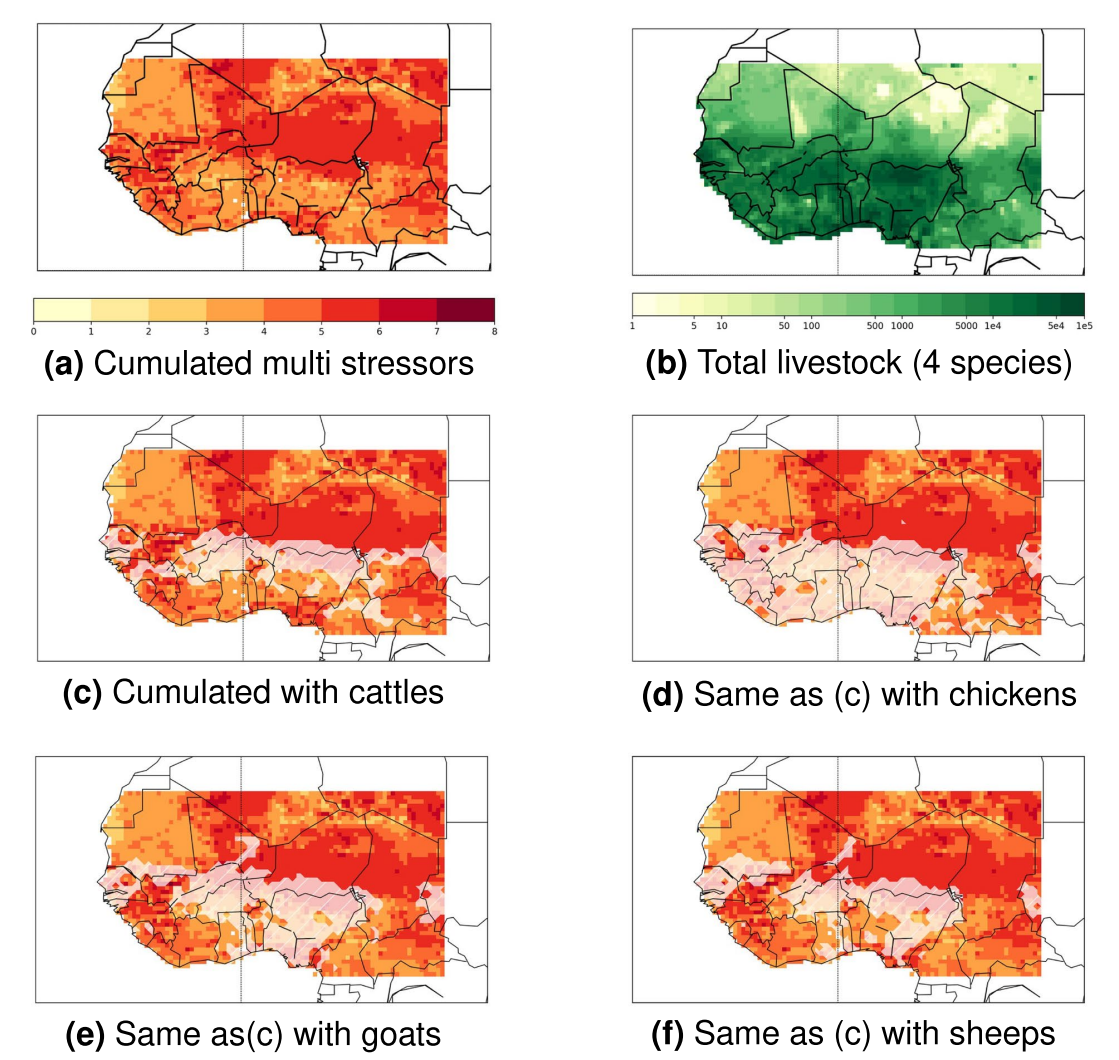
Spatial distribution of cumulated multiple stressors and livestock exposure. The cumulated significance is obtained from the addition of each of the eight stressor indicator spatial distributions that gives a value of 1 if the impact future change is $\ge$ than one standard-deviation (std) calculated over the impact values in the 1979–2005 period, and a value of 0 if not (Figure S4). A value of 8 here indicate 8 combined significant future changes of the stressors, whereas a 0 indicate no significant stressor changes. White areas indicate grid points where there is at least 1000 heads of the corresponding livestock (absolute livestock numbers are displayed in Figure S5).

Each indicator future change is normalized by its corresponding historical standard-deviation (std) calculated over annual values (Figure S4 and S5) to build a spatial distribution of cumulated multiple stressors (see detailed *Methods*). An ”hotspot” pattern is found in north-eastern part of the region, with at least 5 over 8 cumulated climate stressors projected to significantly intensify (Fig. 2a). This area mainly covers Mali, Niger, Chad and Western Sudan. Most of other areas are characterized by a minimum of 4 future cumulated stressors, mostly explained by the three THI indicators that are projected to significantly increase all over Western Africa (Figure S6). Annual mean LAI future adverse change is significant in the northern and western regions, and flood metrics (rainy days and high surface runoff) in the north-eastern part (Figure S6). As the spatial distribution is analysed for projected adverse changes, significant and cumulated projected *positive* future changes are also displayed in Figures S7 and S8. For these change signs, only LAI and low extreme runoff over the eastern part of West Africa are projected to positively change in the future, mainly driven by a corresponding projected regional wettening (Fig. 1).

In addition, the spatial distribution of the eight main livestock species provided by the Gridded Livestock of the World (GLW3^30^) exhibits the largest livestock density located in the south-west of Western Africa (Fig. 2b), oppositely to the north-eastern part of the region for the highest number of cumulated stressors (Fig. 2a). In south-eastern Mali, Niger and in western Sudan, areas with at least 5 cumulated stressors future intensification coincide with high livestock densities (60,000–100,000 heads). Along these 10–12$^{\circ }$N latitudes, species spatial extends show a colocalisation of large densities of the four main livestock species: cattle, chicken, goat and sheep (Fig. 2c–f, Figure S1).

These spatial correlated areas emphasize a 8,468,183 total exposed animals to 5 cumulated stressors, i.e. 30$\%$ of total Western African livestock. This exposed livestock includes 827,738 cattle (i.e. 35$\%$ of total cattle in Western Africa), 4,690,045 chicken (26.5$\%$), 1,510,089 goat (37$\%$), and 1,149,923 sheep (38$\%$). For few grid points, livestock are even projected to be exposed to 6 over 8 multiple stressors increases, with a total of 460,032 animals (i.e. 1.6$\%$) including 39,189 cattle (1.7$\%$), 251,155 chicken (1.4$\%$), 81,838 goat (2$\%$) and 65,224 sheep (2.2$\%$).

**Figure 3:**
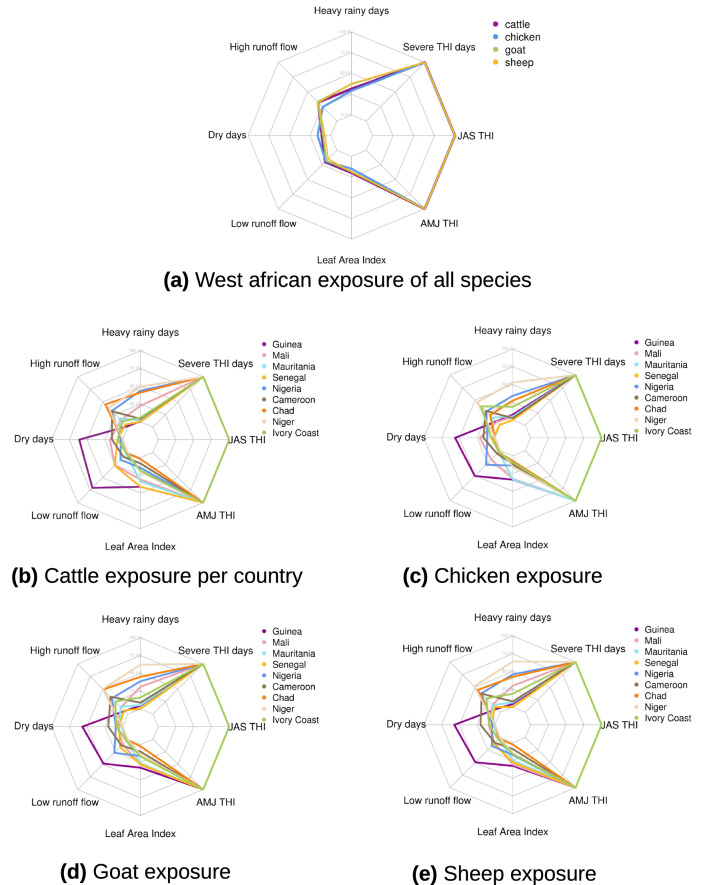
Radarcharts of exposed livestock species to each of the eight selected climate stressors for (**a**) all Western Africa, and (**b**–**e**) per country (in $\%$ of total number of animals per species). Exposure is calculated for each indicator as the sum of all grid points where future projected change is at least equal to the corresponding historical standard-deviation as described in the Methods section.

All livestock species in Western Africa will be concerned by significant heat stress increase, but small exposure to adverse changes in consecutive dry days, low runoff flow, and LAI under the highest warming level (Fig. 3). However, some differences for other stressors between species can be detected. As an example, chicken show a different exposure compared to cattle, goat and sheep populations. As chicken show higher exposure to more consecutive dry days compared to the three other main species, it exhibits a smaller exposure to risks in heavy rainy days, high runoff flow and LAI (Fig. 3a). This difference can be explained by the relative co-location of cattles, goats and sheeps, oppositely to chicken that are more homoneously spatially distributed in West Africa (Figure S2, Fig. 2c–f).

Large differences are also exhibited amongst livestock species per country (Fig. 3b–e). Some countries such as Nigeria and Mali are characterized by similar exposures among all the four main livestock species, whereas specific location/species combinations result in most severe exposures. As an example, 50 to 75$\%$ of all the four main livestocks are projected to experience increasing risk in consecutive dry days days and low runoff flow in Guinea, whereas no more than 25$\%$ of each species will experience such risk in any of other West African countries (Fig. 3b–d). Niger is characterized by 50 to 60 $\%$ of exposed livestock to risks in heavy rainy days and high runoff flow, with a smaller exposure for cattle (< 40$\%$). These livestock exposures quantifications in Guinea and Niger both are consistent with future projected drying (wettening) pattern in west (east) of West Africa (Fig. 1e–g, Figure S3). Senegal correspond to the assessed country with the smallest livestock exposure to cross-sectoral stressors (other than heat stress future intensification) in West Africa, and is characterized by mean species exposure smaller than 15$\%$ except for adverse LAI changes.

### Timing and concurrence of cumulated stressors

**Figure 4:**
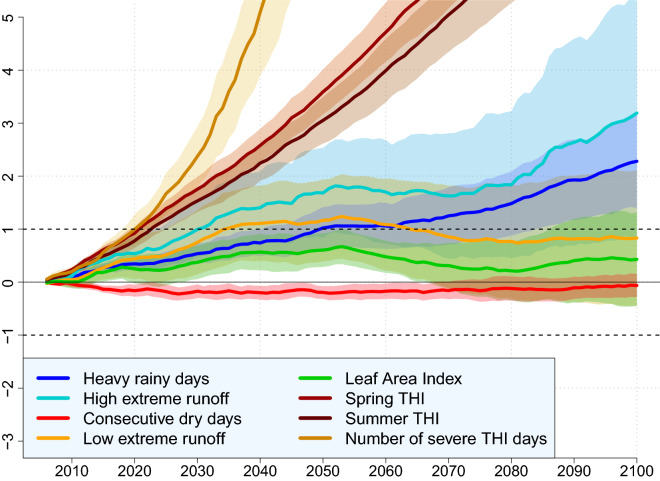
Timeseries of the eight selected climate stressors between 2005 and 2100 averaged over grid points where at least 5 of them are projected to significantly intensify under the RCP8.5 scenario. For each indicator and for each year, values are obtained by dividing the corresponding 20-y upcoming projected change compared to the 1979–2005 period by the 1979–2005 standard-deviation (std). Solid lines indicate the multi-model means for each indicator. Shaded colors display the confidence interval among the models for each impact indicator (see Table S1 for models set for each indicator).

For each climate stressor, we quantify when the first projected mean 27-year difference with averaged 1979–2005 values will be significant according to the models (i.e first year y when $\Delta _{y, y+27}\ge \text{std}_{1979-2005}$). Regional means are then calculated over grid points with at least 5 cumulated projected stressors (Fig. 2a) to extract the corresponding first year when a future change is significant. Heat stress (THI) indicators are the first stressors to reach their associated historical inter-annual variability (i.e. standard-deviation; std) in 2023, mainly due to the warming rate under the RCP8.5^23^ (Fig. 4). An exponential timeseries illustrates the severe heat stress days evolution due to combined very small historical std (Figure S4) and fast THI intensification. Flood metrics are also characterized by a future evolution larger than their respective historical std, from 2031 for the high extreme runoff increase, and then from 2050 for very heavy rainy days (Fig. 4). Annual mean LAI is projected to increase and consecutive dry days to slightly decrease between 2005 and 2050 (i.e. ”positive” changes), but none of these changes reach their corresponding historical std by the end of the century. These timeseries are consistent with the total of 5 cumulated over 8 various climate stressors highlighted in these regions (Fig. 2). Consistently with the projected wettening of eastern West Africa (Fig. 1), low extreme runoff also shows a significant projected positive change (i.e. decreasing drought risk) between 2035 and 2063, but that does not remain significant until 2100 (Fig. 4).

These timeseries depict the spread accross the models, which varies depending on the climate indicator. High and low runoff extremes exhibit large inter-model uncertainties, whereas more direct climate metrics such as THI indexes or very heavy rainy days are characterized by smaller intermodel spread (Fig. 4). This is consistent with previous studies that show how both GIMs and ESMs uncertainties cumulate in multi-model simulations, and how GIMs spread tend to be the largest uncertainties component in climate impact simulations^31,32^.

## Discussion

This study provides a unique assessment of how multiple climate-related stressors that cover major impact sectors may cumulate and co-occur during the 21st century in West Africa. Bias-corrected projections from CMIP5 and ISIMIP2b are analysed to quantify the regional livestock exposure to these cumulated changes, with associated per-species and per-country levels analysis. Under the RCP8.5 and according to climate and impact models, we estimate that:

- A large part of West Africa including Mali, Chad, Niger and Sudan affected by at least 5 to 6 cumulated multiple climate stressors during the 21st century;
- One third of the total western african livestock will be exposed to these cumulated 5–6 stressors, including sheep, cattle, chicken and goat (between 26$\%$ and 38$\%$ of each species regional distribution);
- This exposed livestock will be first affected by a significant intensification of severe heat stress conditions from early 2020s, then by large flood risk from the following decade.

In this paper, the multi-model spread and global impact models (GIMs) vs Earth System Models (ESMs) uncertainties are also investigated by accounting for different GIMs and ESMs datasets for each climate indicator. Previous studies have shown that the majority of GIMs underestimate the extremeness of impacts in heat-related mortality, agriculture and terrestrial ecosystems, while impacts on water resources and hydropower are over-estimated in some river basins both with a large spread among the models^32,33^. These combined uncertainties from future scenario and multi-model must be taken into account when interpreting our cumulated multiple stressors, and our study here confirms the need for taking into account uncertainties and improving model representations for next generations of climate and impact models (CMIP6, ISIMIP3).

Results presented in this paper are based on the RCP8.5 scenario, which assumes that the current high greenhouse gases emissions baseline will continue until at least 2100^34^. Previous works has shown that RCP8.5 high emissions result in a future continued increase in vulnerability of food security, particularly across vulnerable regions such as West Africa^16,35^. They have also shown that even high levels of adaptation investment may not be sufficient to counteract these large emissions associated impacts on food security. Though lower emissions future scenarios project smaller impacts, differences in future changes between different RCPs are not pronounced by until 2050^36^. Since our results show significant intensifications of at least five multiple stressors between 2021 and 2050 (Fig. 4), these results would not be modified under other RCPs. After 2050, and since negative effect becomes more pronounced under RCP8.5^35^, implications of our cumulated multiple stressors may be dampened under mitigated scenarios (e.g. RCP2.6, RCP4.5). The RCP8.5 scenario is also debated as a plausible future due to the needed coal availability to fit along with high greenhouse gases emissions^37,38^.

This paper quantifies the livestock exposure to future cumulated stressors through the use of the static GLW3 livestock spatial distribution for the year 2010^30^. Livestock data thus does not provide either seasonal dynamic/displacements of animals nor future projected livestock evolution, which might modulate our results. Previous studies have shown an expected decline of 7.5 to 9.6$\%$ of global 2010’s livestock under the RCP8.5 scenario due to decreasing herbaceous production, with highest impact projected within sub-Saharan western Africa^39^. Here we support this increasing risk trend for livestock during the 21st century, and argue that multiple stressors may add to smaller net primary production and worsen the projected livestock decline.

We address questions about how increases in multiple stressors could cumulate and result in stronger climate risks during the 21st century. Nevertheless, proper impacts on animals husbandry are not quantified in the present study, neither what stressor and/or impact have the strongest effect on livestock. Previous studies have shown that a decline of -0.099 kg milk/day per THI unit can be measured for cattle under pastoral management systems during *moderate* heat stress days^22,40,41^. As a first preliminary approximation, the effect of (1) *severe* heat stress days that only includes one over the five future cumulated climate stressors shown in corresponding areas, on (2) dairy cattle that are one of the four main affected species can still be quantified. In these regions, adverse intensifications will (at least) enhance the projected decline of milk production by − 8.9 kg milk/day per year from 2021 within Western Africa (Figs. 1c and 4). Poultry birds are also affected by strong heat events that result in increased morbidity and mortality, lowered egg hen-day production, and reduction in feed consumption of at least 1.5$\%$ per increase degree of *moderate* heat stress^42^. Though we did not find any evidence of ”a worst to a better” ranking of impacts on livestock and thus of climate future stressors, literature suggest that hot extremes and associated feedbacks are the main source of adverse impacts for animal husbandry^43^. This statement is particularly verified in Western Africa where extreme events are expected to amplify^5,13^, concurrently to low adaptive capacity^44^.

Our work mainly focus on the magnitude and the cumulative aspect of multiple stressors increases between the end of the 20th and the 21st century. Timing questions are slightly explored, and a further study about emergence and possible co-emergences of multiple stressors or impacts would bring insights about the severity of combined changes for local populations of people and livestock^27,45^. Multiple indicator seasonal cycles are also independently considered, although possible synchronous occurrences and compound events may result in amplified risks^46^ for livestock. Limited bias-corrected atmospherical variables to calculate some of the stressors limit the spatial extent of our study, but focusing on other regions would attribute a larger context to possible severe consequences here emphasized for west african animal husbandry.

The cumulative approach highlights a possible severe future multi-intensification of various climate stressors according to CMIP5 and ISIMIP2b models. However, both resilience and adaptation of livestock systems could reduce and/or counteract these adverse cumulated stressor intensifications^44^. Current literature suggest a large set of various adaptation strategies for animal husbandry to climate change, both for animals and livestock management by helders^16,35,47,48^. These possible adaptations include—but are not limited to—keeping two to three species of livestock simultaneously on the same farm to obtain sustainability and economic benefits; using better adapted livestock breeds and species; adjusting watering point to future changes of spatial and seasonal patterns of forage production; more effective matchings of stocking rates with feed production. Their effects may depend on the level of the projected warming.

Some of these livestock adaptations might also mitigate greenhouse gas emissions^8^. Indeed, current livestock production accounts for 16–22$\%$ of total greenhouse-gas emissions and contributes to climate change and its adverse cross-sectoral consequences at a global scale^49^. The growth of livestock farms to fit with the increasing food demand also intensifies water needs, particularly due to waters needs to grow crops used for animals feeding^48^. In our work, induced stressors of global warming on the livestock farming is investigated but we do not assess these livestock feedbacks on resulting risks. A relevant further work would be also to assess how livestock adaptation strategies may contribute themselves to animal husbandry exposures and vulnerabilities (e.g. livestock displacements may results in local farmers/herdsmen conflicts^50,51^).

## Methods

### Model simulations and climate forcings

The Inter Sectoral Impact Model Intercomparison Project (ISIMIP)^20^ consists in a cross-sectoral synthesis of multiple impacts of climate change and includes associated uncertainties. The specific ISIMIP2b protocol is selected in our study since it provides impact simulations from 1950 to 2100 for many sectors such as global water, climate extremes, agriculture, health, energy supply and demand or regional forests^20^. It also enables a good representation of mean climate forcings and variabilities from the 5th version of the Coupled-Models Intercomparison Project (CMIP5^19^). In ISIMIP2b, four climate models were indeed selected to represent mean CMIP5 responses needed as inputs data for impact models of each sector: two high-climate sensitivity models (i.e. IPSL-CM5A-LR and HadGEM2-ES) and two low-climate sensitivity models (i.e. MIROC5 and GFDL-ESM2M). Three main Representative Concentration Pathways (RCPs) that illustrates possible greenhouse gases future scenarios are also available and include RCP2.6, RCP6.0 and RCP8.5^20^. In this paper, analyses are focused on the highest emissive RCP8.5 greenhouse-gases scenario, since it provides the largest/clearest signal of the global warming on the multiple climate-related stressors we assess. Further details on ISIMIP2b specific protocol, data acquisition and bias-correction are provided in Frieler et al.^20^ and Lange^52^. Note that raw variables to calculate the eight indicators are not simulated by the same GIMs-ESMs combinations, resulting in a different total number of simulations for each indicator. Detailed General Impact Models (GIMs) and Earth System Models (ESMs) combinations are displayed in Table S1.

### Livestock data

The livestock exposure to multiple stressors is quantified using the Gridded Livestock of the World (GLW3^30^) from the Food and Agriculture Organization (FAO). This dataset provides gridded data of absolute livestock quantity (i.e. number of heads) at a global scale for the year 2010 for eight main species: cattle, chicken, goat, sheep, pig, duck, horse and buffaloe (Figure S1). Thought these data do not provide future projections of livestock, livestock dynamics and diurnal/seasonal displacements, it enables a robust assessment of a fixed livestock exposure to future cross-sectoral and combined stressors over Western Africa due to its high spatial resolution and multiple represented species.

### Climate-related stressors

In this paper, a stressor correspond to a negative evolution of a climate-related indicator that may adversely affect livestock. Previous studies have shown that global warming could affect the livestock system by various ways, including water ressources, food availability or climate extremes^16^. To fairly represent these major sectors, we assess corresponding indicators similarly as in Piontek et al.^3^ and Byers et aL.^4^ about future impacts, by combining future projected changes in these sectors that may cumulated and/or co-occur, and thus strongly stress livestock species in Western Africa.

#### Food availability

Access to food is a major sector affecting livestock living conditions and productivity^8^. As grazing corresponds to the main feed source for digastric grazers (e.g. cattle, sheep, goat), analysing the variability of vegetal cover provides an indicator of changes in the food availability for animals husbandry. In this work, the Leaf Area Index (LAI) is considered as a proxy of vegetation productivity due to its wide used as a basic vegetation descriptor^53–55^. However, LAI should not be considered as a direct feeding source for all the eight livestock analysed due to their feeding system (e.g. vegetal species diversity, grains for poultry)^16^. LAI can be defined as the amount of leaf area in $\text{m}^2$ per unit ground area in $\text{m}^2$, and allows to gauge the amount of photosynthesizing biomass. As a dimensionless quantity, LAI can be measured and simulated at a global scale, and thus provides an essential indicator of crop development and growth. This variable is simulated in ISIMIP2b both for historical and RCP8.5 scenarios, and for a large set of GIMs and ESMs (see Table S1).

#### Drought risk

As direct (hydration) and indirect (feed quality) impacts, water scarcity strongly affects livestock livelihood^16^. Drier and hotter conditions increase water needs of plants and animals, particularly in regions already water stressed such as Western Africa. Regionally, water scarcity results from a complex feedback and enhancing/dampening equilibrium between extreme hot temperatures, lack of soil moisture and lack of precipitation^56^. Droughts may also decrease water quality for animal consumption through increased polluants, pathogens or salts in water. Drought metrics were thus developped for land-atmosphere feedbacks and physical understanding, as well as for impact analyses. In previous works, the magnitude of the 5$\%$ lowest daily runoff per year (values $\le$ 5th percentile) were defined as an indicator of low flow^57^. According to ISIMIP2b data availability^58^, we similarly analyse the yearly lowest 2$\%$ daily total (i.e. surface + subsurface) runoff of each year (values $\le$ the 2th percentile), and here consider this metric as a *low flow* index and a drought risk indicator. In addition, the number of consecutive days with daily precipitation smaller than 3mm per year is quantified (here after *consecutive dry days*), to strenghten drought metrics analyses^59^.

#### Flood risk

Flood data strongly depend on the available data, which rely on local flood statistics, regional flood statistics, rainfall-runoff modelling, and local recommended choice^60–62^. Flood studies usually require runoff data, river catchment data and case study of real flood events. These data are then compared to flood simulations to investigate needed conditions to exhibit possible flood event depending on regional conditions. Due to ISIMIP2b data availability and with the purpose to analyse the highest number of combined GIMs $\times$ ESMs simulations, we assess the highest daily runoff per year as a flood risk index in this paper. As for droughts, an indicator of yearly *high flow* is defined as the highest 5$\%$ daily runoff values per year^57^. Similarly, the annual highest 2$\%$ daily runoff (values $\ge$ 98th percentile) is calculated and considered as a *high flow* index and thus a flood risk indicator. In addition, we quantify the number of days per year when daily precipitations are higher than 20mm, referenced here after as a *very heavy rainy days* indicator^59^.

#### Heat stress

The Temperature-Humidity Index (THI)^21^ is here analysed as a common used indicator of heat stress on livestock. THI incorporates both high surface temperature and relative humidity (RH) effects to describe the level of heat stress on livestock^22^. This index is often used in impact studies since THI values can be interpreted in terms of proper risks and consequences for livestock. As an example, THI values above 89 are considered as *severe* heat stress conditions and may result in an increase in respiration rate, excessive saliva production, dry matter intake decrease, and productive and reproductive decrease^40^. For THI values higher than 98, heat stress is very dangerous and livestock may die.

The original formula incorporates RH and the temperature of the dry bulb (Tdb). However, due to the lack of both observed and simulated Tdb, equations were later adjusted and approximated using the daily air temperature instead of the Tdb^22,40^. In this study, we analyse the highest daily heat stress to assess the greatest likely risk for livestock species. It results in the use of the daily maximum temperature (Tmax) instead of the daily mean temperature in THI calculation, similarly as previous studies^22,63^. Daily RH is usually derived from temperature and surface vapor pressure data in CMIP5 and ISIMIP2b, and we here consider the accuracy of the calculated RH at the moment of Tmax (i.e. $\text{RH}_{{max}}$) under the hypothesis that surface specific humidity does not signficantly change during the day^22^. THI daily values can be calculated as:

1 $$\begin{aligned} THI = (1.8 \times Tmax + 32) - [ (0.55 - 0.0055 \times RH_{max} \times (1.8 \times Tmax - 26.8) ] \end{aligned}$$

with THI the Temperature-Humidity Index^21,22^, Tmax the daily maximum temperature, and $\text{RH}_{{max}}$ the corresponding daily relative humidity when Tmax. Details of intermediate calculation steps are provided in Supplementary Materials.

### Cumulative approach of multiple stressors

To investigate how these various climate change indicators may cumulate in the future, each projected change is normalized and added to obtain a spatial distribution of combined climate stressors. For each stressor indicator, its projected change between 1979–2005 and 2074–2100 absolute value is divided by its corresponding historical standard-deviation (std) calculated over 1979–2005 values. A spatial mask is then created, with 1 where this normalization gives values $\ge$ 1, and 0 when normalised change < 1. By adding each 1/0 climate stressor masks, a spatial distribution where each grid point is characterized by a value between 0 (no significant climate-related stressor) and 8 (all stressor changes are significant and cumulate) is thus obtained. Corresponding results are displayed in Fig. 2 and Figure S5. As described in the “Results” section, this additive approach is weightened with the sign of each climate stressor projected change.

It is important to note that each climate-related indicator is considered here as additive with a misleading assumption of independency of one another. Yet, most of these stressors are correlated in time and space, and the assumed independance in the cumulated index has no physical meaning. We argue this approximation to assess how combined stressors for a same location may adversely affect local animal husbandry (e.g. heat stress increase for the two consecutive warmest seasons of the year). All selected indicators here illustrate how livestock will experience intensifications for consecutive and cumulated multiple climate stressors.

## Supplementary Information

Supplementary Information.

## Data Availability

Raw datasets analysed in this work can be found at https://esg.pik-potsdam.de/search/isimip/ for ISIMIP2b impact data and at http://amma2050.ipsl.upmc.fr/ for CMIP5 bias-corrected climate data.
