# Supplementary material for: Livestock exposure to future cumulated climate-related stressors in West Africa
Source: Sci Rep. 2023 Feb 15;13:2698. doi: 10.1038/s41598-022-22544-y (PMC9932094; doi:10.1038/s41598-022-22544-y)
Supplement: Supplementary file 1 — Supplementary Information. [file 41598_2022_22544_MOESM1_ESM.pdf]

SUPPLEMENTARY MATERIALS - Livestock exposure to future cumulated climate-related stressors in West Africa

Audrey Brouillet[1] **and Benjamin Sultan**[1]

[1]ESPACE-DEV, Univ Montpellier, IRD, Univ Guyane, Univ Reunion, Univ Antilles, Univ Avignon, Montpellier. France

This document contains analyses information about model features, detailed calculations of the heat stress indicator, and supplementary figures referenced in the main paper. Content of this file:

- Model features (Table S1)
- Calculation of the Temperature-Humidity Index (THI)
- Figures S1 to S8

Model features

GIMs data (ISIMIP2b) all include time-varying human influences across the historical period (i.e. *histsoc* simulations), except for CLM45 in which human influences for the historical scenario are fixed at present-day (*2005soc*)[1]. For RCP8.5 future scenario, human influences are fixed at present-day for all GIMs. Note that ISIMIP2b data all follow bias-correction steps as described in Lange et al. [2018][3].

To account for CMIP5 multi-model spread and to be consistent with the four climate inputs represented in ISIMIP2b (i.e. GFDL-ESM2M, HadGEM2-ES, IPSL-CM5A-LR and MIROC5), 10 ESMs from CMIP5 were selected to calculate Temperature-Humidity Index (THI) indicators, very heavy rainy days and consecutive dry days. ESMs data were all bias-corrected to get timeseries from 1950 to 2099 on a $0.5^\circ \times 0.5^\circ$ regular grid over Africa[4]. Model bias correction has been performed using the cumulative distribution function-transform (CDF-t) method[5], by matching the CDF of a simulated climate variable to the CDF of this variable in observations through a mathematical transformation. The observation-based reference is the WATCH Forcing Data methodology applied to ERA-Interim data WFDEI[6]. More details about bias correction steps can be found in Vrac et al. [2012][7] and Famien et al. [2018][4].

Impact indicators	Raw data	Simulations	Model names
**Very heavy rainy days**<br>**Consecutive dry days**<br><br>**Temperature-Humidity Index** indicators (3)	daily precipitation during May-September ($kg.m^{-2}.s^{-1}$)<br><br>daily maximum temperature (°C) + surface specific humidity ($kg.kg^{-1}$) + sea-level pressure (hPa)	10 ESMs i.e. 10 simulations (CMIP5)	ACCESS1-0<br>BNU-ESM<br>CanESM2<br>CSIRO-Mk3-6-0<br>GFDL-ESM2M<br>HadGEM2-ES<br>IPSL-CM5A-LR<br>MIROC5<br>MRI-ESM1<br>NorESM1-M
**High runoff flow**<br>**Low runoff flow**	daily runoff during May-September ($kg.m^{-2}.s^{-1}$)	5 GIMs × 4 ESMs i.e. 20 simulations (ISIMIP2b)	H08<br>LPJML<br>MATSIRO<br>ORCHIDEE<br>WATERGAP2<br><br>GFDL-ESM2M<br>HadGEM2-ES<br>IPSL-CM5A-LR<br>MIROC5
**Leaf Area Index**	monthly leaf area index of all plant functional type (no unit)	3 GIMs × 4 ESMs i.e. 12 simulations (ISIMIP2b)	CLM45<br>ORCHIDEE<br>VISIT<br><br>GFDL-ESM2M<br>HadGEM2-ES<br>IPSL-CM5A-LR<br>MIROC5

Table 1. ISIMIP2b and CMIP5 model characteristics for each of the eight impact indicators analysed in the main paper. The highest number of combined Global Impact Models (GIMs) × Earth System Models (ESMs) simulations both for historical and RCP8.5 scenarios was chosen per indicator, depending on the available data. It results in the here mentionned models combination per impact indicator. Descriptions and features of the different models can be found in Frieler et al. [2017][1] for GIMs and Taylor et al. [2012][2] for ESMs.

Temperature-Humidity Index (THI) calculations:

The Temperature-Humidity Index (THI[8]) incorporates the effects of both high surface relative humidity (RH) and surface air temperature, to describe the level of heat stress on livestock[9]. It can be calculated as :

$$THI = (1.8 \times Tmax + 32) - [(0.55 - 0.0055 \times RH_{max}) \times (1.8 \times Tmax - 26.8)] \quad (1)$$

with THI the Temperature-Humidity Index[8,9], Tmax the maximum daily temperature, and RH_{max} the associated daily relative humidity. RH_{max} is not provided in CMIP5 but can be calculated using RH calculation steps and by replacing usual T by Tmax in following intermediate calculations:

$$RH = \frac{VP}{VPsat} \times 100 \quad (2)$$

with RH in %, VP the air vapor pressure (in hPa), and VPsat the saturation vapor pressure (in hPa) calculated from: VPsat=6.11×exp(0.067×T), with T in °C (Buck et al. (1996)). Note that this VPsat formula only works for surfaces with no ice cover. The air vapor pressure VP can be calculated as:

$$VP = \frac{q \times psurf}{0.622 + 0.378 \times q} \quad (3)$$

with q the surface specific humidity (in $kg_{water}/kg_{humid.air}$) and psurf the surface air pressure (in hPa). Psurf is not directly provided as daily CMIP5 simulations, and is thus derived from the sea-level pressure using hydrostatic equilibrium and assuming adiabatic conditions as:

$$psurf = psl \times \exp\left(\frac{-g \times z}{r \times T}\right) \quad (4)$$

with psurf and psl in hPa. g is the gravitational constant (9.81 m/s^{-2}), r the gaz constant of the air with a value of 287.067046 J/kg/K (obtained from dividing the molar gas constant R (i.e. 8.3144321 J/mol/K) with the molar mass of the air (i.e. 29 g/mol). T is the surface air temperature in K, and z the altitude in meters.

References

1. Frieler, K. *et al.* Assessing the impacts of 1.5 °C global warming – simulation protocol of the Inter-Sectoral Impact Model Intercomparison Project (ISIMIP2b). *Geosci. Model. Dev.* **10**, 4321–4345, DOI: 10.5194/gmd-10-4321-2017 (2017).
2. Taylor, K. E., Stouffer, R. J. & Meehl, G. A. An Overview of CMIP5 and the Experiment Design. *Bull. Am. Meteorol. Soc.* **93**, 485–498, DOI: 10.1175/BAMS-D-11-00094.1 (2012).
3. Lange, S. Bias correction of surface downwelling longwave and shortwave radiation for the EWEMBI dataset. *Earth Syst. Dyn.* **9**, 627–645, DOI: 10.5194/esd-9-627-2018 (2018).
4. Famien, A. M. *et al.* A bias-corrected CMIP5 dataset for Africa using the CDF-t method – a contribution to agricultural impact studies. *Earth Syst. Dyn.* **9**, 313–338, DOI: 10.5194/esd-9-313-2018 (2018).
5. Michelangeli, P.-A., Vrac, M. & Loukos, H. Probabilistic downscaling approaches: Application to wind cumulative distribution functions. *Geophys. Res. Lett.* **36**, L11708, DOI: 10.1029/2009GL038401 (2009).
6. Weedon, G. P. *et al.* The WFDEI meteorological forcing data set: WATCH Forcing Data methodology applied to ERA-Interim reanalysis data. *Water Resour. Res.* **50**, 7505–7514, DOI: 10.1002/2014WR015638 (2014).
7. Vrac, M. *et al.* Dynamical and statistical downscaling of the French Mediterranean climate: uncertainty assessment. *Nat. Hazards Earth Syst. Sci.* **12**, 2769–2784, DOI: 10.5194/nhess-12-2769-2012 (2012).
8. National Research Council & Committee on Physiological Effects of Environmental Factors on Animals. *A guide to environmental research on animals* (National Academy of Sciences (US), 1971).
9. Rahimi, J., Mutua, J. Y., Notenbaert, A. M. O., Dieng, D. & Butterbach-Bahl, K. Will dairy cattle production in West Africa be challenged by heat stress in the future? *Clim. Chang.* **161**, 665–685, DOI: 10.1007/s10584-020-02733-2 (2020).
10. Gilbert, M. *et al.* Global distribution data for cattle, buffaloes, horses, sheep, goats, pigs, chickens and ducks in 2010. *Sci. Data* **5**, 180227, DOI: 10.1038/sdata.2018.227 (2018).

Figures

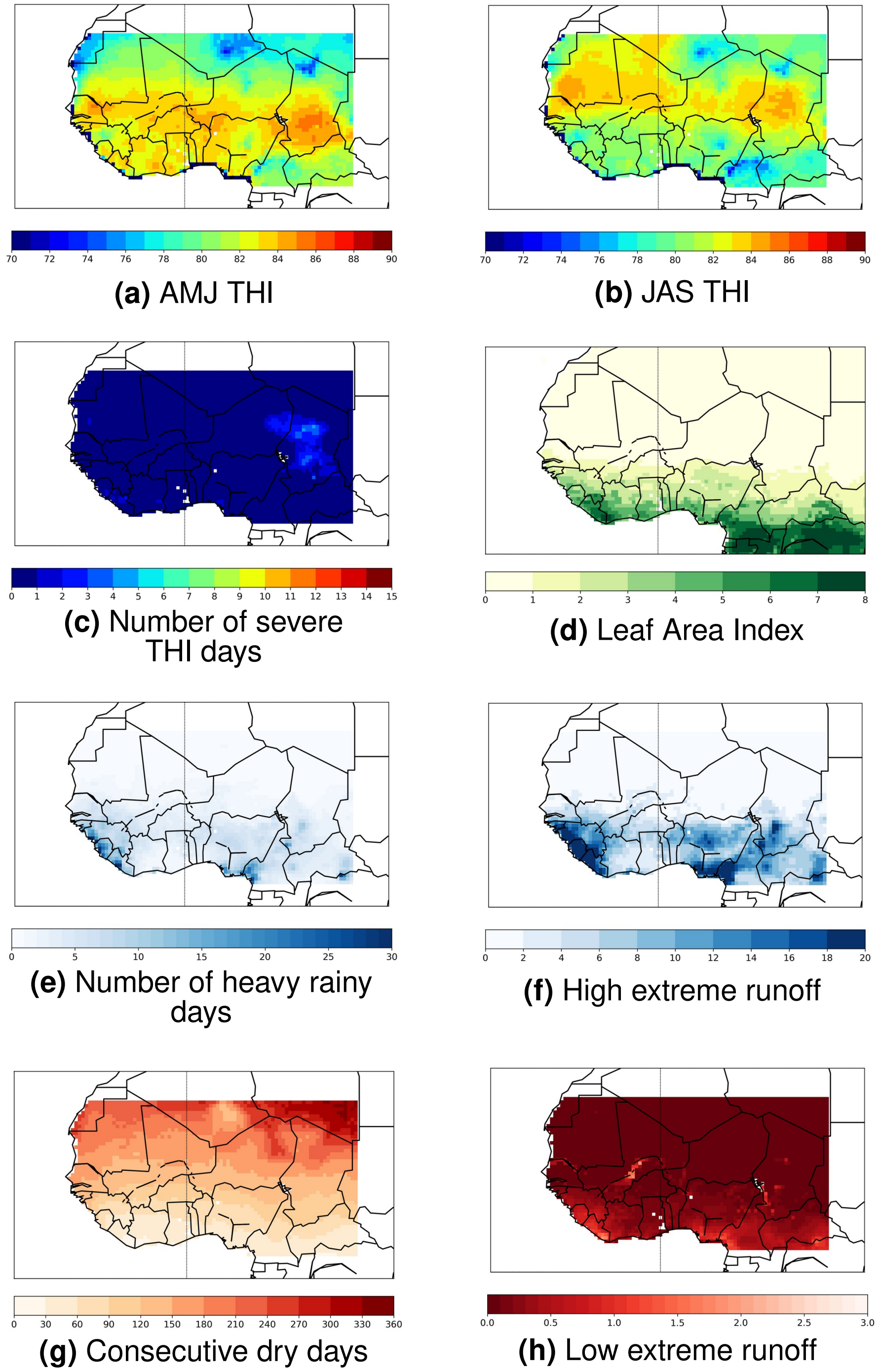

Figure 1. Multi-model mean historical (1979-2005) values for the 8 selected indicators. **(a)** April-to-June mean THI (no unit). **(b)** July-to-September mean THI (no unit). **(c)** Number of days per year with *severe* daily heat stress (i.e. THI $\geq$ 89). **(d)** Leaf Area Index (no unit). **(e)** Number of days during May-September with daily precipitation > than 20mm. **(f)** Annual 98th percentile of surface + subsurface runoff (in mm/day). **(g)** Number of consecutive days during May-September with daily precipitations < than 3mm. **(h)** Annual 2nd percentile of surface + subsurface runoff (in mm/day).

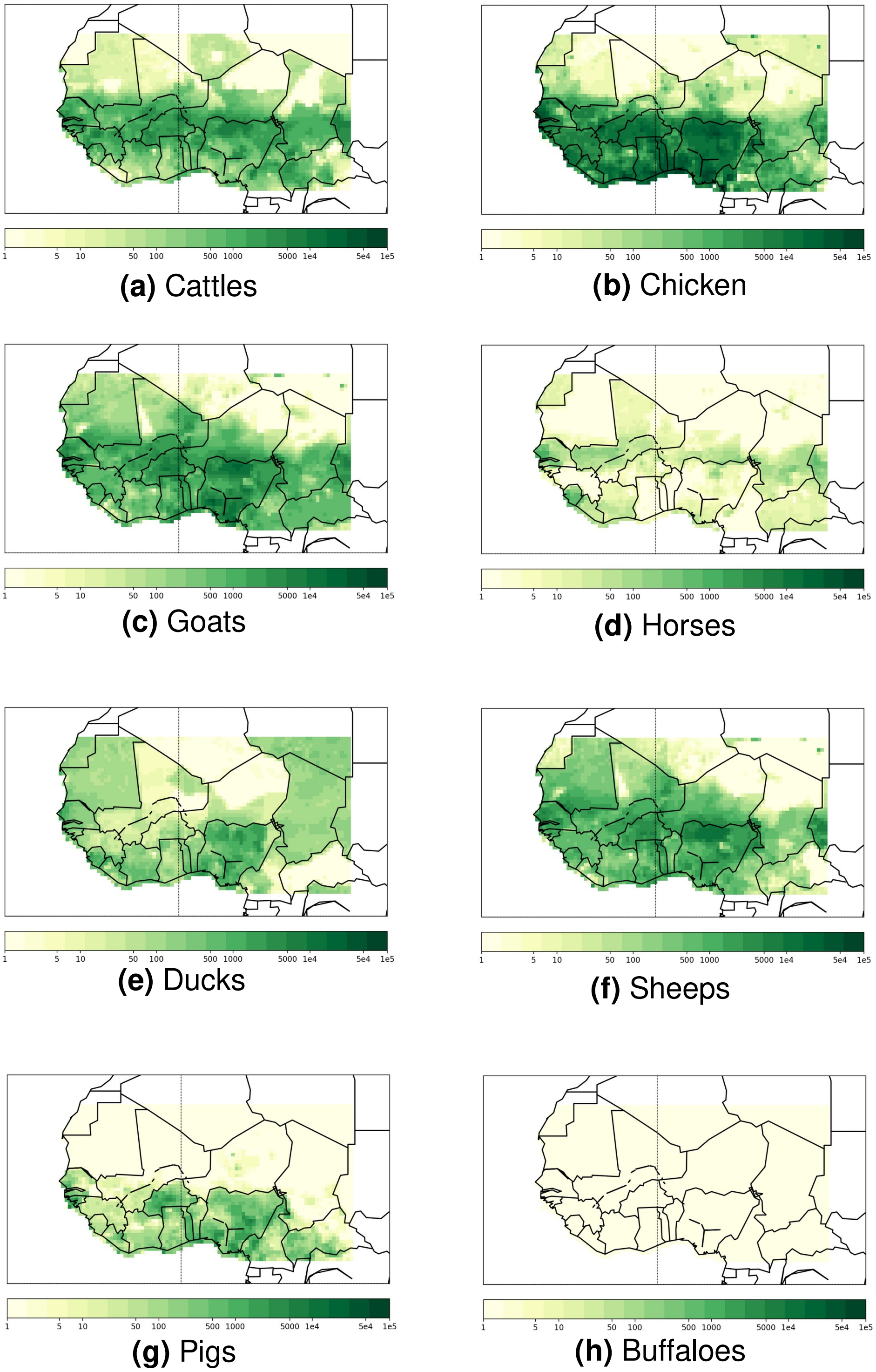

Figure 2. Absolute livestock number of each of the 8 species from in the Gridded Livestock of World (GLW3)[10] at logarithmic scale. GLW3 was first developped for the year 2007 at global scale from the Food and Agriculture Organization (FAO), and was later updated for the year 2010. Due to their relative densities, main paper exposure analyses are mostly conducted on cattles, chicken, goats, ducks, sheeps and pigs.

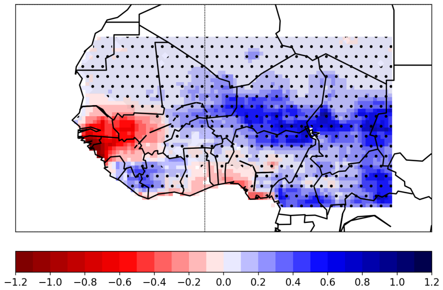

(a) Annual mean precipitation

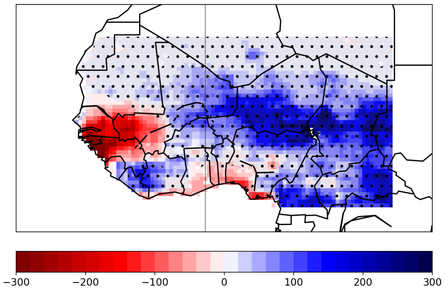

(b) Annual sum of daily precipitations

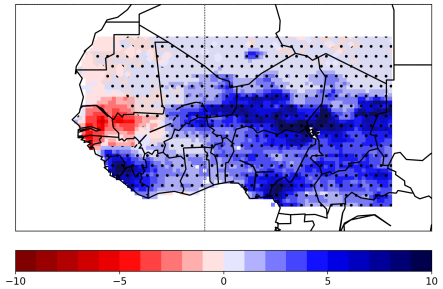

(c) Annual 98th percentile of precipitation

Figure 3. Multi-model mean projected change in precipitation metrics averaged over the four climate input simulations in ISIMIP2b (i.e. IPSL-CM5A-LR, HadGEM2-ES, MIROC5 and GFDL-ESM2M[1,2]. **(a)** Annual mean of daily precipitation (in mm/day). **(b)** Annual sum of all daily precipitations (in mm). **(c)** Annual 98th percentile of daily precipitation (i.e. the 7th highest day). Dots show grid points where at least 50% of the models agree on the sign of the change.

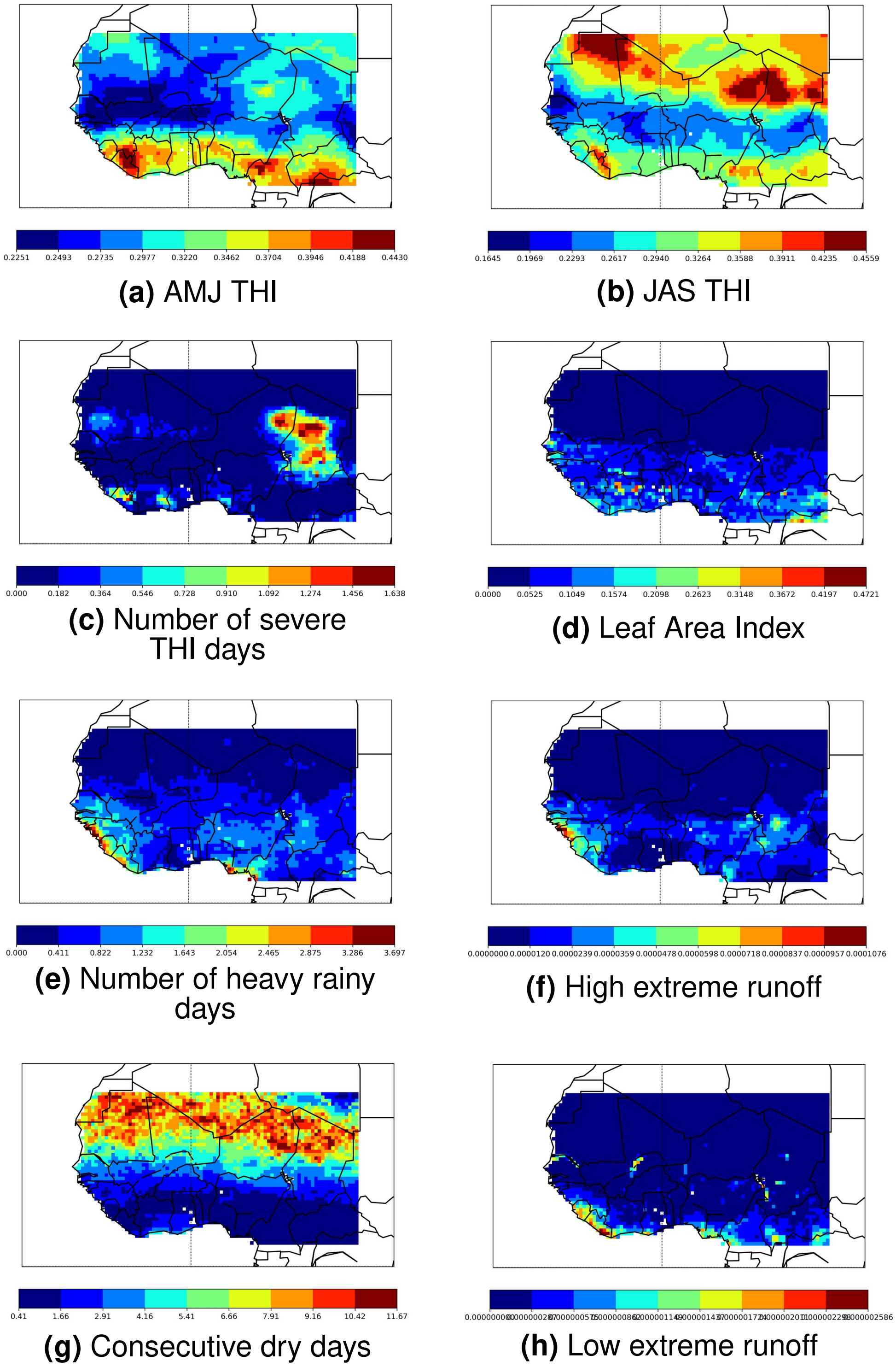

Figure 4. Multi-model mean standard deviation (std) for the 8 selected impact indicators calculated over the 1979-2005 period (no unit). (**a**) April-to-June mean THI. (**b**) July-to-September mean THI. (**c**) Number of days per year with *severe* daily heat stress (i.e. THI $\geq$ 89). (**d**) Leaf Area Index (no unit). (**e**) Number of days during May-September with daily precipitation larger than 20mm (**f**) Annual 98th percentile of surface + subsurface runoff. (**g**) Number of days during May-September with daily precipitations smaller than 3mm. (**h**) Annual 2nd percentile of surface + subsurface runoff.

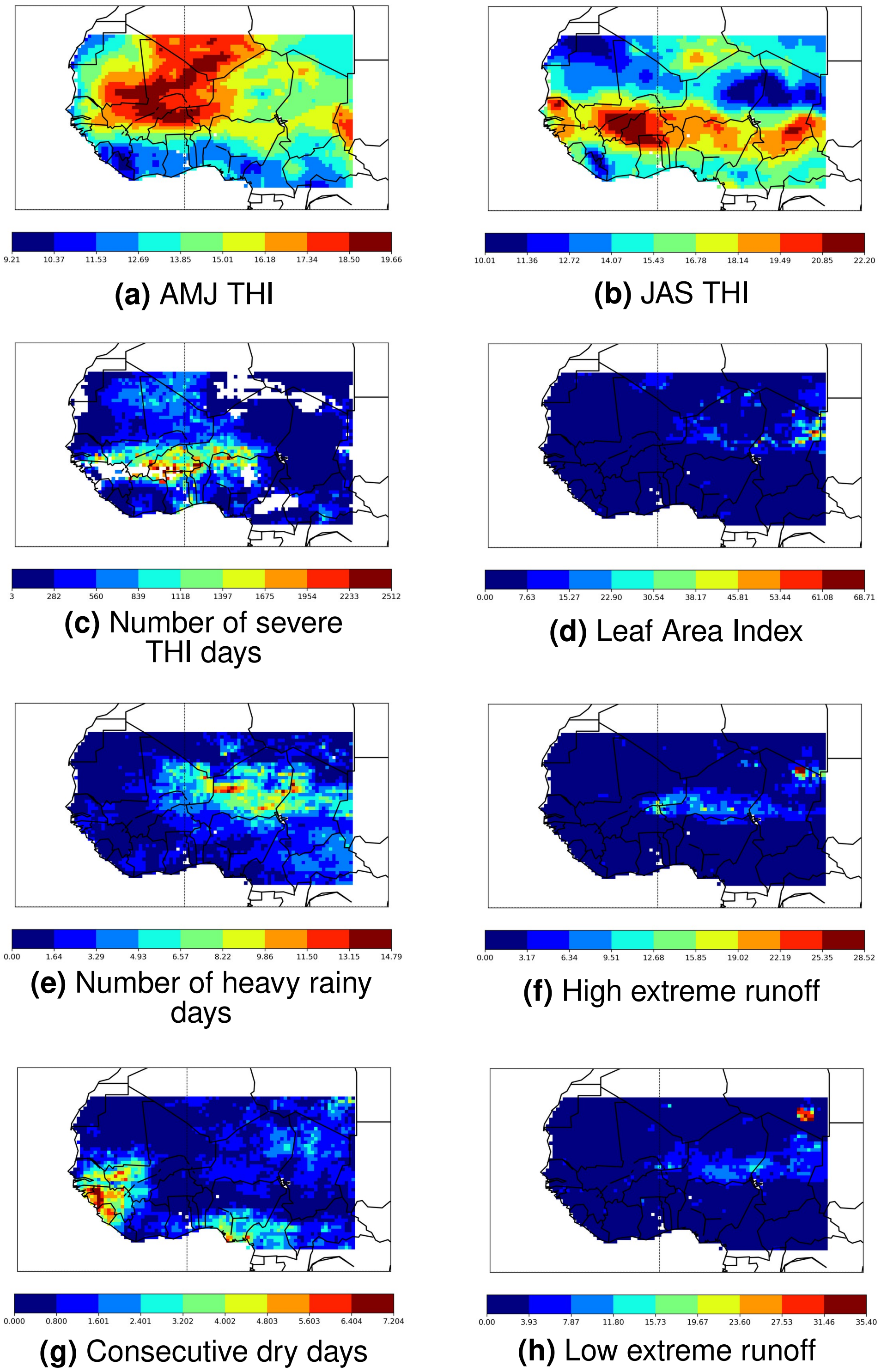

Figure 5. Multi-model mean normalized projected future change by the 1979-2005 std for the 8 selected impact indicators. **(a)** April-to-June mean THI. **(b)** July-to-September mean THI. **(c)** Number of days per year with *severe* daily heat stress (i.e. THI $\geq$ 89). **(d)** Leaf Area Index (no unit). **(e)** Number of days during May-September with daily precipitation larger than 20mm **(f)** Annual 98th percentile of surface + subsurface runoff. **(g)** Number of days during May-September with daily precipitations smaller than 3mm. **(h)** Annual 2nd percentile of surface + subsurface runoff.

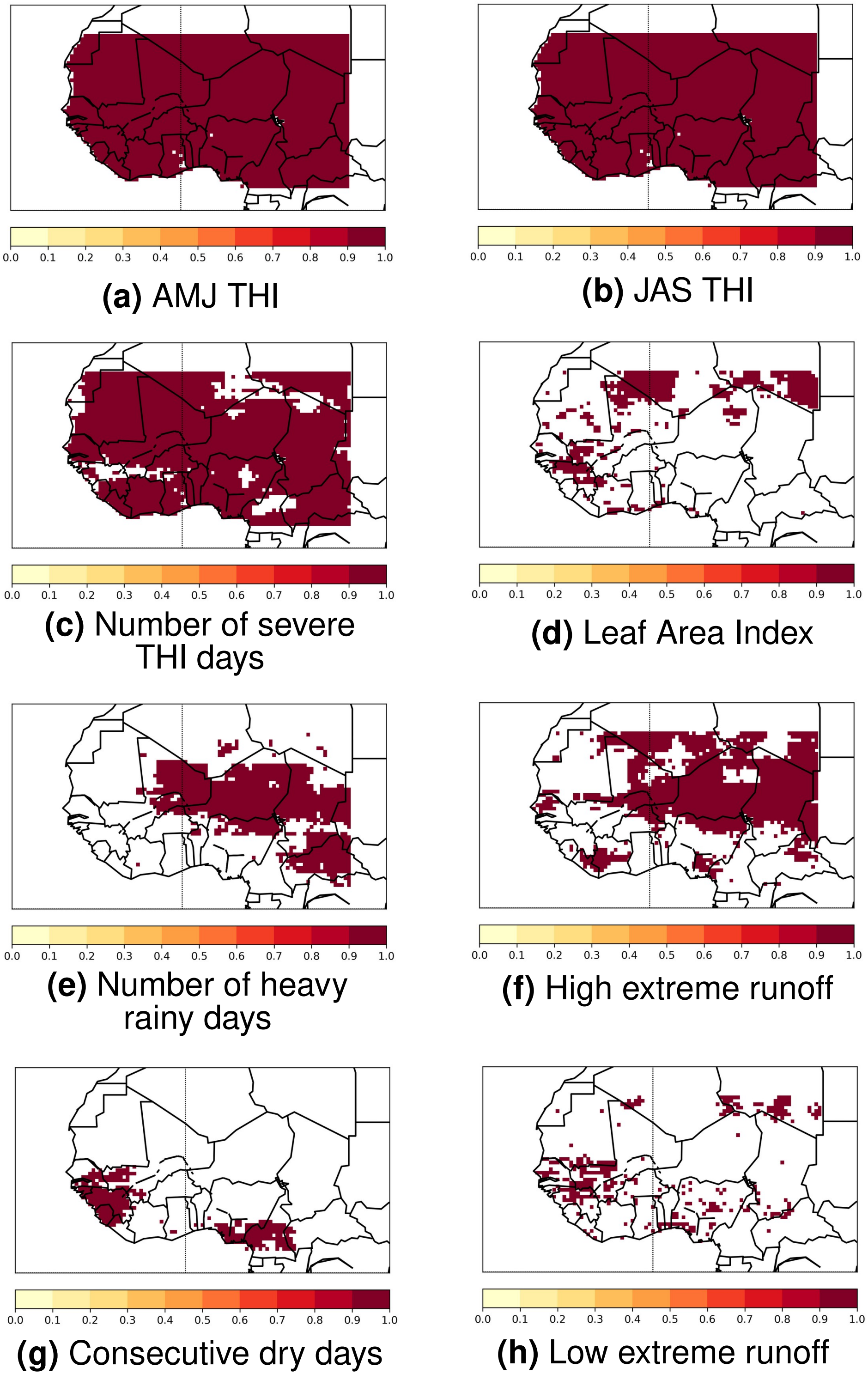

Figure 6. Multi-model mean mask when projected ADVERSE change is higher than one historical std for the 8 selected impact indicators. (**a**) April-May-June THI increase. (**b**) July-August-September THI increase. (**c**) *Severe* THI days increase. (**d**) Leaf Area Index decrease. (**e**) Heavy rainy days increase. (**f**) High extreme runoff increase. (**g**) Consecutive dry days increase. (**h**) Low extreme runoff decrease.

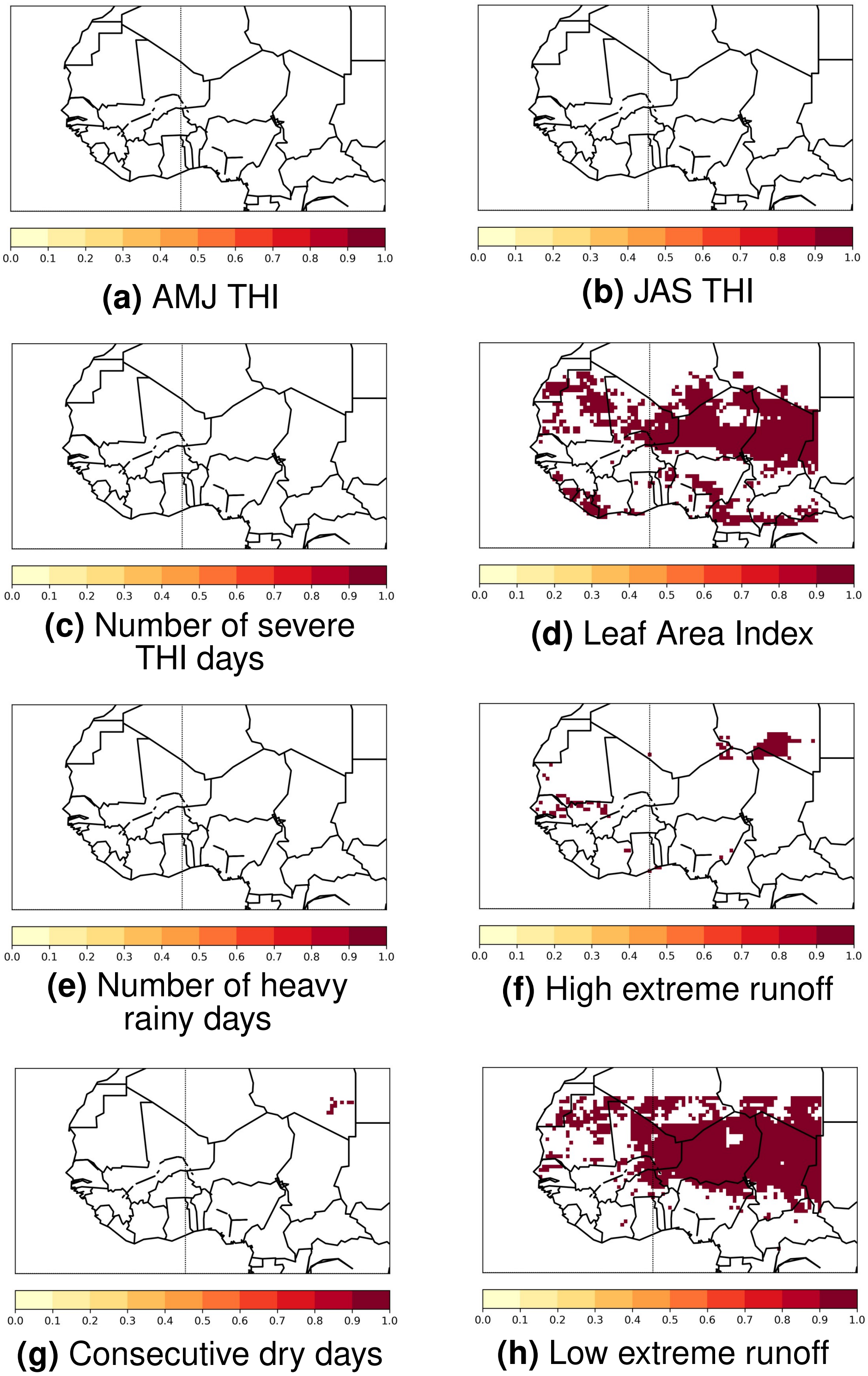

Figure 7. Multi-model mean mask when projected POSITIVE change is higher than one historical std for the 8 selected impact indicators. (**a**) April-May-June THI increase. (**b**) July-August-September THI increase. (**c**) *Severe* THI days increase. (**d**) Leaf Area Index decrease. (**e**) Heavy rainy days increase. (**f**) High extreme runoff increase. (**g**) Consecutive dry days increase. (**h**) Low extreme runoff decrease.

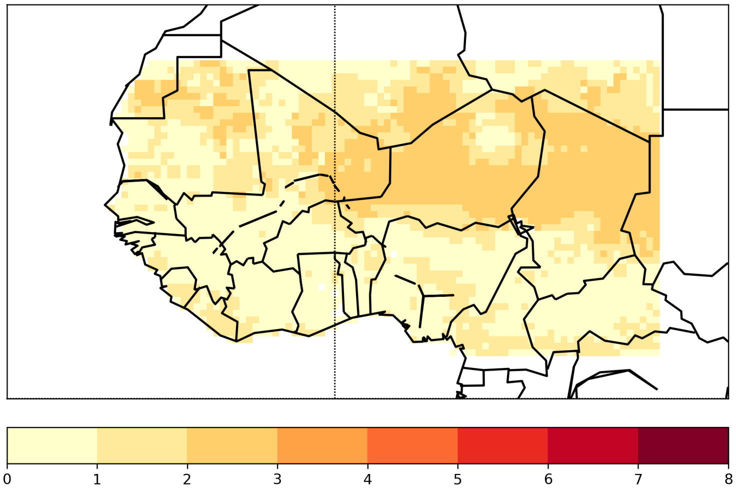

Figure 8. Spatial distribution of cumulated multiple *benefits* (positive projected changes) Same as main paper Figure 2a but with positive changes instead of multiple adverse projected changes.
